# Effects of Financial Expenditure of Prefectures/Municipalities on Regional Suicide Mortality in Japan

**DOI:** 10.3390/ijerph18168639

**Published:** 2021-08-16

**Authors:** Takashi Shiroyama, Kouji Fukuyama, Motohiro Okada

**Affiliations:** Department of Neuropsychiatry, Division of Neuroscience, Graduate School of Medicine, Mie University, Tsu 514-8507, Japan; takashi@clin.medic.mie-u.ac.jp (T.S.); k-fukuyama@clin.medic.mie-u.ac.jp (K.F.)

**Keywords:** suicide mortality, Japan, prefecture, governmental expenditure, welfare

## Abstract

In Japan, suicide mortality has been improving from 2009; however, suicide remains one of the leading causes of death. Although previous studies identified solid relationships between governmental financial support for social welfare systems and suicide mortality, little attention is paid to how specific regional policies, designed according to regional cultural, economic, and social welfare situations, affect suicide mortality. Therefore, the present study analyses the relationships between the regional governmental expenditure of six major divisions and suicide mortality across the 47 prefectures in Japan from 2009 to 2018 using fixed-effect analysis of hierarchal linear regression with robust standard error. The expenditure in “public health”, “police”, “ambulance/fire services”, “welfare” and “education” is associated with reduction in suicide mortality, at least in some statistical indicators, whereas expenditure of “public works” indicated the influence of increasing suicide mortality or had no effect. Welfare expenditure was the most predominantly effective among the six major divisions of regional governmental expenditure. In the welfare subdivisions, expenditure of “child welfare” and “social welfare” was effective in a reduction in suicide mortality, but expenditure of “elderly welfare” surprisingly contributed to increasing suicide mortality. Child welfare expenditure negatively impacted suicide mortality in wide-ranging generations of both males and females; the positive effects of elderly welfare expenditure reached were limited as working-age populations increased, but unexpectedly did not affect the suicide mortality of elderly populations. The relatively increasing expenditure of elderly welfare with the relatively decreasing child welfare are unavoidable due to the Japanese social issues associated with a declining birth rate and ageing population. Furthermore, the budget of that regional government that can modify its expenditure structure by making its own policies is limited since most regional governmental expenditure is composed of essential expenditure for maintaining and operating regional social welfare systems. Although severe social situations in Japan are still unoptimised, the present results suggest that scientific-evidence-based redistributions of welfare expenditure in regional governments can at least partially improve Japanese society and welfare systems.

## 1. Introduction

Suicide is a major global public health concern, since suicide mortality is approximately 800,000, and the annual global-age-standardised suicide rate is 10.53 per 100,000 population [1,2,3,4,5]. Japanese public health concerns associated with suicide are more serious. Suicide victims in Japan rose to more than 30,000 deaths in 1998 and continued for a decade (at a maximum of 40.1 deaths per 100,000 males in 2003) [6,7,8,9]. Following the collapse of the asset bubble in 1991 with the Asian economic crisis in 1997, macroeconomic status in Japan deteriorated, including increasing unemployment rate accompanied by increasing suicide mortality in Japan in 1998. During the early 1990s, suicide mortality of males in Japan was lower than that of European countries (17–18 per 100,000 population); however, in spite of the improving macroeconomic status in Japan between 2003 and 2008, higher incidences of suicide mortality of Japanese males were sustained from 1998 to 2008 [6,10,11,12]. These dynamics of suicide mortality in Japan associated with economic crises revealed that suicide consists of a complicated psychosocial pathomechanism, wherein financial hardships are only one of the putative contributing factors [13]. Against the Japanese public health crisis, the Japanese government enacted several policies, including the Basic Act on Suicide Prevention and the General Policies for Comprehensive Measures against Suicide to organise comprehensive suicide-prevention programmes from 2006.

Japanese governmental financial support for a comprehensive suicide-prevention programme commenced in 2008. Japan also experienced a significant macroeconomic downturn due to the 2008 global financial crisis; however, suicide mortality in Japan was improving in the period 2009–2019. Unfortunately, in 2020, suicide victims increased compared to in 2019, from 20,169 (males: 14,078, females: 6091) to 20,919 (males: 13,943, females: 6976). In spite of a slight increase in total suicide victims (males and females) in 2020, male suicide victims continued to decrease [11]. The decrease in male suicide mortality from 2009 predominantly contributed to the decrease in suicide mortality in Japan rather than that of female mortality (the decrease in female suicide mortality is relatively small compared to that of males) [10,11]. The Ministry of Health, Labour, and Welfare (MHLW) announced an alert seven times to the mass media [14,15] from September 2019 to May 2021, saying that the report should be conducted according to suicide reporting guidelines “Preventing suicide: a resource for media professionals” [16], since the increased suicide mortality in 2020 was speculated to have been induced by the drastic modification of lifestyle caused by the pandemic of coronavirus disease 2019 (COVID-19) and the overheated reports of mass media associated with the suicide and death of celebrities with COVID-19 [17]. Although the detailed mechanisms of the increase in suicide mortality in 2020 still need to be scientifically clarified, the MHLW added a budget for dealing with COVID-19-related apprehensions to the grant for Enhanced Community-Based Suicide Countermeasures [18]. Therefore, the Japanese government provides financial support as a basic response to various types of crises, since it evaluates the usefulness of enhancement of regional social and welfare systems supported by governmental finance.

Several studies have also reported that governmental financial supports for the enhancement of welfare and social security systems contributed to a reduction in suicide mortalities in Europe, the USA, and South Korea [19,20,21,22,23,24]. In Italy, active labour-market and vocational-rehabilitation programmes contributed to a reduction in the suicide mortality of working-age males to compensate for the reduced social integration resulting from unemployment, supporting individuals to continue to integrate into society [21,22]. In South Korea, increased governmental support for the enhancement of social welfare facilities for the elderly population improved suicide mortality in regions with a higher proportion of elderly residents at a high risk of suicide [19]. In the USA, the addition of more states into supplemental nutrition-assistance programmes, which support individuals with low or no income to purchase food, was related to a reduction in suicide mortality [24]. Conversely, the deteriorated provision of regional social welfare services due to severe cuts to regional governmental budgets from 2010 to 2012 is probably related to the highest suicide mortality in this century at 2013 in England [24,25]. From 2010 to 2012, the coalition government in England implemented policies (austerity and welfare reform) to improve public deficits that had drastically increased due to the 2008 global financial crisis [26]. During the recession induced by the 2008 global financial crisis, employment status and other macroeconomic factors in England began to recover in 2011; however, suicide mortality and several mental health issues continued to deteriorate even after economic recovery in England [24,25]. Deteriorated mental health during recessions recovers as employment status is improved [27]. Therefore, in spite of the improvement of the economy, the sustained deterioration of both suicide mortality and mental health in England is considered to possibly be affected by governmental policy and severe cuts of regional governmental budgets (austerity and welfare reform). 

However, little attention is paid to how specific regional policies, designed according to regional cultural, economic, and social welfare situations, affect suicide mortality in Japan. Therefore, to clarify the impact of regional governmental policies on suicide mortality, the present study analysed the relationships between regional governmental expenditure of six major divisions, namely, “public health”, “public works”, “police”, “ambulance/fire services”, “welfare” and “education”, and overall gender- and age-specific suicide mortality across the 47 prefectures in Japan between 2009 and 2018 using fixed-effect analysis of hierarchal linear regression with robust standard error.

## 2. Materials and Methods

### 2.1. Dependent Variables

The data of suicide victims in all 47 prefectures in Japan from 2009 to 2018 were obtained from the national governmental Basic Data on Suicide in the Region (BDSR) database of the MHLW [11]. BDSR publishes annual suicide data on the basis of the following age groups disaggregated by gender: younger than 20 years old (10s), 20–29 (20s), 30–39 (30s), 40–49 (40s), 50–59 (50s), 60–69 (60s), 70–79 (70s), and over 80 years old (80s) [11]. Annual prefectural suicide mortality is calculated by dividing suicide mortality per prefecture by the prefectural population (denominator) of the same years, obtained from the Regional Statistics Database: System of Social and Demographic Statistics of the Statistics Bureau of the Ministry of Internal Affairs and Communications (SBMIAC) [28]. To eliminate small prefectural population artifacts, prefectural suicide mortality was calculated by using the empirical Bayes standardised mobile ratio method by using the empirical Bayes estimator for the Poisson/gamma model (v2.1) (National Institute of Public Health, Wako, Japan) (https://www.niph.go.jp/soshiki/gijutsu/download/ebpoig/index_j.html (Access on 14th August 2021) [29]. Annual standardised death rates for the suicide mortality (SDR) of males, females and both together were calculated on the basis of the Japanese age-dependent population composition in 2009 for males and females. 

### 2.2. Independent and Dependent Variables

Regional financial expenditure was obtained from the Survey of Local Public Finance Settlement (SLPFS) from SBMIAC [30,31]. The regional governmental expenditure for inhabitant services is mainly composed of the expenditure of six divisions, public health, public works, police, ambulance/fire services, welfare, and education [30,31]. The present study adopted the expenditure of these six divisions per capita and the ratio of the expenditure of six divisions against the total amount of the regional governmental expenditure, published in SLPFS, as independent variables [32].

The public health expenditure of prefectures and their municipalities is composed of support for or the improvement of medical care, mental health, and collecting and disposing of general waste, in order to maintain and improve public health and the regional environment [32]. 

The public works expenditure of prefectures and their municipalities is composed of the construction of new public facilities and the improvement of existing ones, including roads, bridges, parks, and sewers [32]. 

The police expenditure of prefectures is composed of the salaries of police officers, police construction, projects, and traffic signals in order to carry out police administration to prevent crime, ensure traffic safety, maintain the safety and order of regional communities, and protect the lives and property of individuals [32].

The expenditure of ambulance/fire services of municipalities is composed of the salaries of fire-station officers, station construction, and fire trucks and ambulances in order to protect individuals from disasters, for emergency cases, and the sudden deterioration of health [32]. 

The education expenditure of prefectures and municipalities is composed of the construction of new or the improvement of existing educational facilities, and the salaries of educational officers. Subdivisions of education expenditure consist of social education (for social education facilities including public halls, libraries, and museums), elementary school education (for the elementary school expenditure of municipalities), junior high school education (for the junior high school expenditure of municipalities), senior high school education (for the senior high school expenditure of prefectures), special school education (for schools for the education of special needs individuals of prefectures) and kindergarten school education (for kindergarten schools of prefectures and their municipalities) [32].

The welfare expenditure of prefectures and municipalities is composed of spending on social welfare, elderly welfare (welfare for individuals 65 years old and over), child welfare (welfare for individuals younger than 17 years old), livelihood welfare (welfare for livelihood-protection individuals) to enhance the social welfare system [30,31]. Social welfare includes welfare for individuals with disabilities and welfare assistance for unclassifiable subjects.

In SLPFS, in the published expenditure per capita of six divisions, subdivisions of social welfare and social education were calculated by dividing their expenditure by the prefectural population (denominator). Expenditure per capita of the subdivisions of welfare, elderly welfare, child welfare and livelihood welfare was calculated by dividing expenditure by populations (denominator) of individuals older than 65 years old, younger than 18 years old and assisted by livelihood protection, respectively. Expenditure per capita of the subdivisions of education, kindergarten, elementary, junior high, and senior high schools was calculated by dividing expenditure by the number of students in schools [32].

### 2.3. Statistical Analysis

The present study analysed the effects of expenditure of six major divisions and the subdivisions of welfare and education on suicide mortality, disaggregated by attributes of gender and age in Japan by fixed effects for prefectures using hierarchical linear regression with robust standard error (HLM7, Scientific Software International, Skokie, IL, USA). Additionally, the present study adopted robust standard errors clustered by prefectures to prevent heteroscedasticity and autocorrelation [8]. 

The trends of suicide mortality and regional financial expenditure in Japan from 2009 to 2018 were analysed by a lineal mixed model using BellCurve for Excel v.3.2 (Social Survey Research Information Co., Ltd., Tokyo, Japan). When the data did not violate the assumption of sphericity (*p* > 0.05), the F value of the lineal mixed model was analysed using sphericity-assumed degrees of freedom. However, if the assumption of sphericity had been violated (*p* < 0.05), the F value was analysed using Chi-Muller’s corrected degrees of freedom. Lastly, when the F value was significant, data were analysed by Tukey’s multiple-comparison test [33,34,35,36].

## 3. Results

### 3.1. Trends of Suicide Mortality and Regional Financial Expenditure in Japan between 2009 and 2018

**Figure 1:**
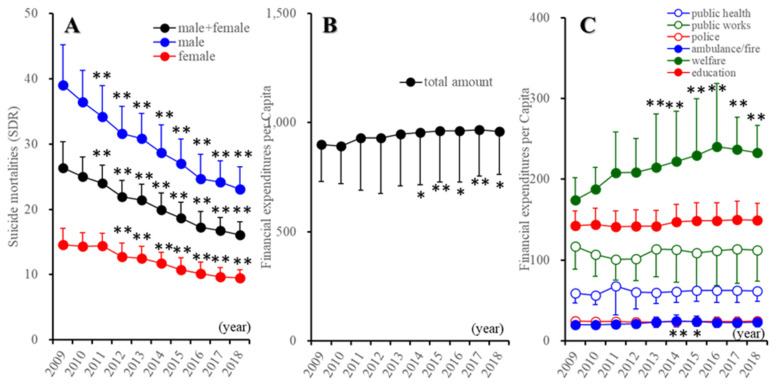
Trends of suicide mortalities (**A**), regional financial expenditure (**B**,**C**) in Japan from 2009 to 2018. (**A**) Ordinate, suicide mortalities per 100,000 population of males and females together, males, and females. (**B**,**C**) Ordinates, regional financial expenditure of prefecture and municipality (JPY 1000 per capita). * *p* < 0.05, ** *p* < 0.01: relative to 2009 by linear mixed model with Tukey’s post hoc test.

From 2009 to 2019, the Japanese suicide mortality of males and females together (F(6.3, 291.2) = 274.4 (*p* < 0.01)), male (F(7.2, 331.4) = 217.9 (*p* < 0.01)) and females (F(7.1, 326.6) = 121.7 (*p* < 0.01)) was significantly decreased time-dependently (Figure 1A). The total amount of the regional financial expenditure of prefectures and their municipalities, on the other hand, increased from 2014 to 2018 compared with that in 2009 (F(1.6, 75.5) = 5.1 (*p* < 0.05)) (Figure 1B). Regarding the regional financial expenditure of six divisions, welfare expenditure increased from 2013 to 2018, compared with that in 2009 (F(1.8, 81.8) = 19.6 (*p* < 0.05)) (Figure 1C). The expenditure of ambulance/fire services was temporarily increased from 2014 to 2015 compared with in 2009 (F(3.1, 141.6) = 41.6 (*p* < 0.01)) (Figure 1C). The expenditure of public health, public works, and education was not changed (Figure 1C); however, the relative proportion of public-works expenditure in the total amount of regional financial expenditure decreased (F(2.8, 127.6) = 13.8 (*p* < 0.01)) (data not shown).

### 3.2. Effects of Total Amount of Financial Expenditure per Capita of Prefectures and Municipalities on Suicide Mortality

**Figure 2:**
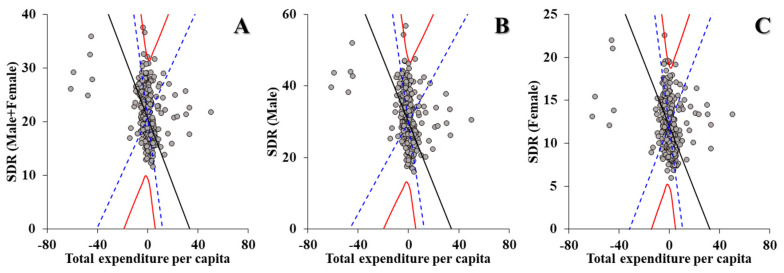
Correlation between total amount of the financial expenditure per capita of prefectures and municipalities, and suicide mortality of males and females together (**A**), males (**B**), and females (**C**) from 2009 to 2018 using fixed- or random-effect analysis of hierarchal linear regression with robust standard error. Ordinates and abscissas, standardised death rates for suicide mortality based on Japanese age-dependent population composition in 2009 (SDR) of males and females together, males, and females per 100,000 population, and centralised total amount of financial expenditure per capita of prefectures and municipalities (JPY 10,000), respectively. Black, blue, and red lines, slope of fixed effects, standard deviation of fixed effects, and fixed effects with random effects, respectively.

The hierarchical linear-regression model detected significant or negative relationships between the total amount of the financial expenditure per capita of prefectures and municipalities and the regional suicide mortality of males and females together (*p* < 0.01), males (*p* < 0.01), and females (*p* < 0.01) (Figure 2). Therefore, an increase in regional financial expenditure (prefectures and municipalities) probably contributed to a reduction in suicide mortality in Japan.

### 3.3. Effects of Relative Ratios of Financial Expenditure of Six Divisions of Prefectures and Municipalities on Suicide Mortality (Model 1)

**Figure 3:**
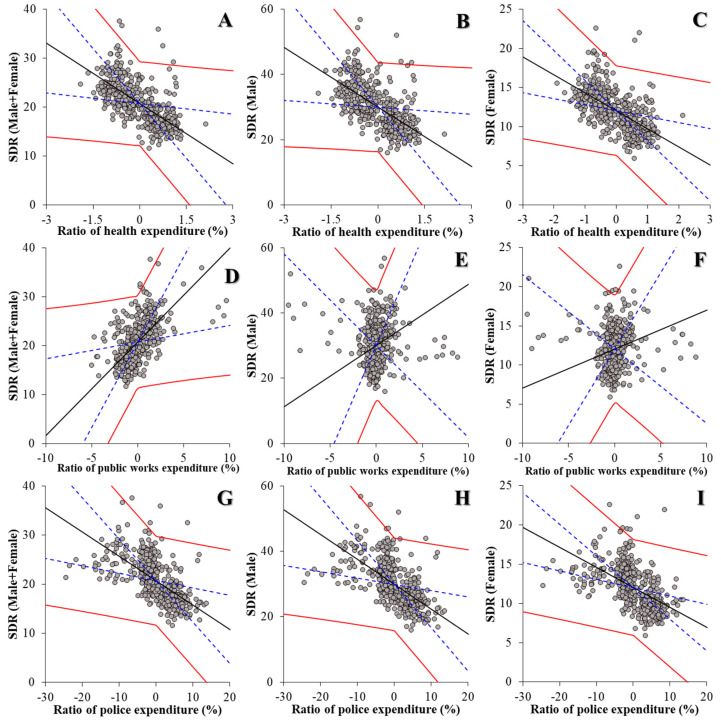
Effects of financial expenditure ratio of public health (**A**–**C**), public works (**D**–**F**), and police (**G**–**I**) of prefectures and municipalities on suicide mortality of males and females together (**A**,**D**,**G**), males (**B**,**E**,**H**), and females (**C**,**F**,**I**) in 2009–2018 using fixed- or random-effect analysis of hierarchal linear regression with robust standard error. Ordinates and abscissas, SDR of suicide mortality (per 100,000 population) and centralised ratios of expenditure per capita of prefectures and municipalities (JPY 10,000), respectively. Black, blue, and red lines, slope of fixed effects, standard deviation of fixed effects, and fixed effects with random effects, respectively.

The hierarchical linear-regression model detected statistically significant relationships between the financial expenditure ratios of prefectures and municipalities, and suicide mortality (Figure 3 and Table 1). The financial expenditure ratios of both health and police were negatively related to the suicide mortality of males and females together, males, and females (Figure 3 and Table 1), whereas the financial expenditure ratio of public works was positively related to the suicide mortality of males and females together, males, and females (Figure 3 and Table 1). These results suggest that increases in the relative health and police expenditure reduce suicide mortality, but an increase in the relative expenditure of public works probably elevates suicide mortality. The other financial expenditure ratios of ambulance and fire services, welfare, and education do not contribute to suicide mortality (Table 1). 

### 3.4. Effects of Financial Expenditure per Capita of Divisions and Subdivisions of Prefectures and Municipalities on Suicide Mortality

#### 3.4.1. Effects of Regional Financial Expenditure per Capita of Six Divisions of Prefectures and Municipalities on Suicide Mortality (Model 2)

**Figure 4:**
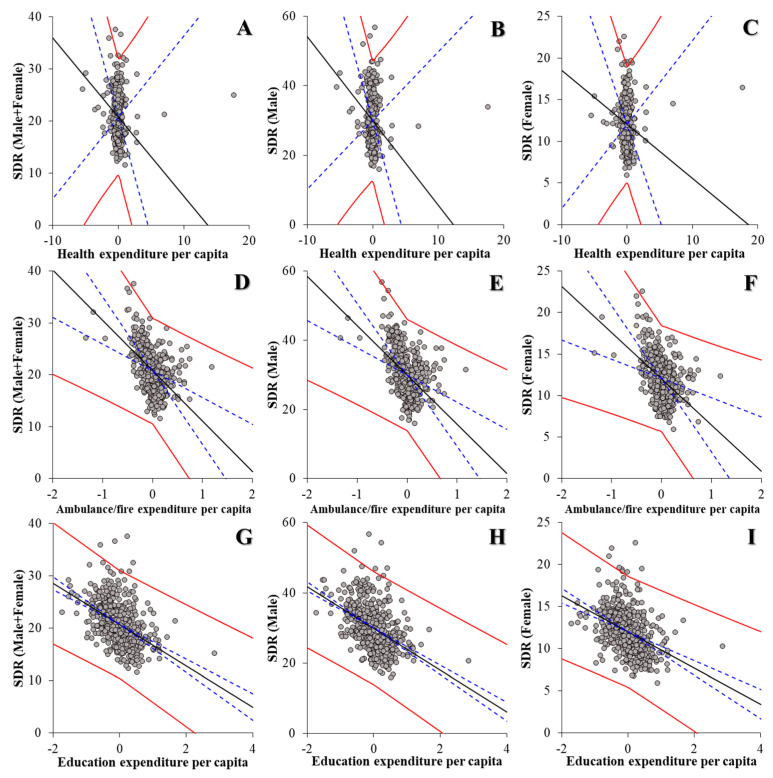
Effects of financial expenditure per capita of public health (**A**–**C**), ambulance and fire services (**D**–**F**), and education (**G**–**I**) of prefectures and municipalities on regional suicide mortality of males and females together (**A**,**D**,**G**), males (**B**,**E**,**H**), and females (**C**,**F**,**I**) from 2009 to 2018 using fixed- or random-effect analysis of hierarchal linear regression with robust standard error. Ordinates and abscissas, SDR of suicide mortalities (per 100,000 population) and centralised expenditure per capita of prefectures plus municipalities (JPY 10,000), respectively. Black, blue, and red lines, slope of fixed effects, standard deviation of fixed effects, and fixed effects with random effects, respectively.

The hierarchical linear-regression model detected negative relationships between the financial expenditure per capita of prefectures and municipalities, and suicide mortality (Figure 4 and Table 2). The financial expenditure of public health, ambulance and fire services, and education was negatively related to the suicide mortality of males and females together, males, and females (Figure 4 and Table 2). These results suggest that the increases in expenditure per capita of public health, ambulance and fire services, and education reduce suicide mortality, whereas the financial expenditure of public works, police, and welfare probably does not contribute to suicide mortality (Table 2). 

#### 3.4.2. Effects of Regional Financial Expenditure per Capita of Divisions and Education Subdivisions on Suicide Mortality (Model 3)

**Figure 5:**
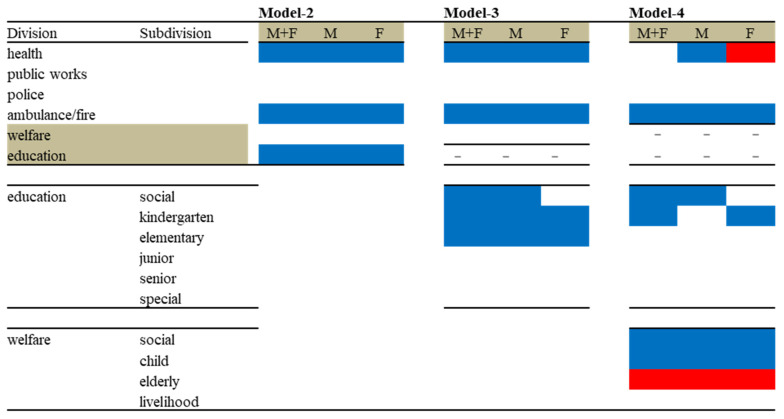
Summary of effects of regional financial expenditure of divisions and subdivisions of education and welfare in prefectures and municipalities on the suicide mortality of males and females together (M + F), males (M), and females (F) from 2009 to 2018. Blue and red columns, significant factors for decreasing and increasing suicide mortality, respectively.

The SLPFS publishes subdivisions of education expenditure per capita [30,31]. Therefore, to explore the more detailed effects of education expenditure on suicide mortality, the replacement of the independent variable, education expenditure, into six subdivisions of education expenditure was analysed with a hierarchical linear-regression model. The hierarchical linear-regression model detected similar relationships between Models 2 and 3 (Figure 5). The financial expenditure of health, and ambulance and fire services was negatively related to the suicide mortality of males and females together, males, and females (Figure 5). The hierarchical linear-regression model also detected negative relationships between the subdivisions of education expenditure of prefectures and municipalities, and suicide mortality (Figure 5). Kindergarten and elementary school expenditure was negatively related to the suicide mortality of males and females together, males, and females (Figure 5). Social education expenditure was negatively related to the suicide mortality of males and females together and males alone, but not to that of females (Figure 5).

#### 3.4.3. Effects of Regional Financial Expenditure per Capita of Divisions and Subdivisions of Education and Welfare on Suicide Mortality (Model 4)

The SLPFS also publishes subdivisions of welfare expenditure [30,31]. Models 1–3 did not detect a significant effects of welfare expenditure; however, Model 3 results showed the importance of more fragmented analysis. Therefore, to explore more detailed effects of welfare expenditure on suicide mortality, the independent variable in Model 3, welfare expenditure, was replaced by four subdivisions of welfare expenditure, and they were analysed by hierarchical linear-regression model.

Contrary to Model 3, the hierarchical linear-regression model detected clearly different effects of the financial expenditure of prefectures and municipalities between Models 3 and 4 (Figure 5). Regarding welfare-subdivision expenditure, the expenditure of both social and child welfare was negatively related to the suicide mortality of males and females together, males, and females, whereas the expenditure per capita of livelihood welfare was not related to suicide mortality at all (Figure 5). Contrary to our expectation, expenditure of elderly welfare was positively related to the suicide mortality of males and females together, males, and females (Figure 5).

Regarding the subdivisions of education expenditure, in Model 3, elementary school expenditure was related to the suicide mortality of males and females together, males, and females. In Model 4, the negative effects of elementary school expenditure were not detected (Figure 5). Similarly, in Model 3, kindergarten school expenditure was related to the suicide mortality of males and females together, males, and females, whereas in Model 4, the negative effects of kindergarten school expenditure on suicide mortality of males were not present (Figure 5). 

Regarding the expenditure of divisions, the expenditure of ambulance and fire services was also negatively related to the suicide mortality of males and females together, males, and females in Model 4; however, surprisingly, public health expenditure was negatively related to the suicide mortality of females in Models 2 and 3; in Model 4, it was positively related to that of females (Figure 5). This discrepancy between male and female responses removed the relationship between public health expenditure and the suicide mortality of males and females (Figure 5). 

### 3.5. Effects of Financial Expenditure of Divisions and Subdivisions per Capita of Education and Welfare on Suicide Mortality Disagregated by Age

#### 3.5.1. Effects of Regional Financial Expenditure of Divisions and Subdivisions of Education per Capita on Suicide Mortality Disaggregated by Age (Model 5)

**Figure 6:**
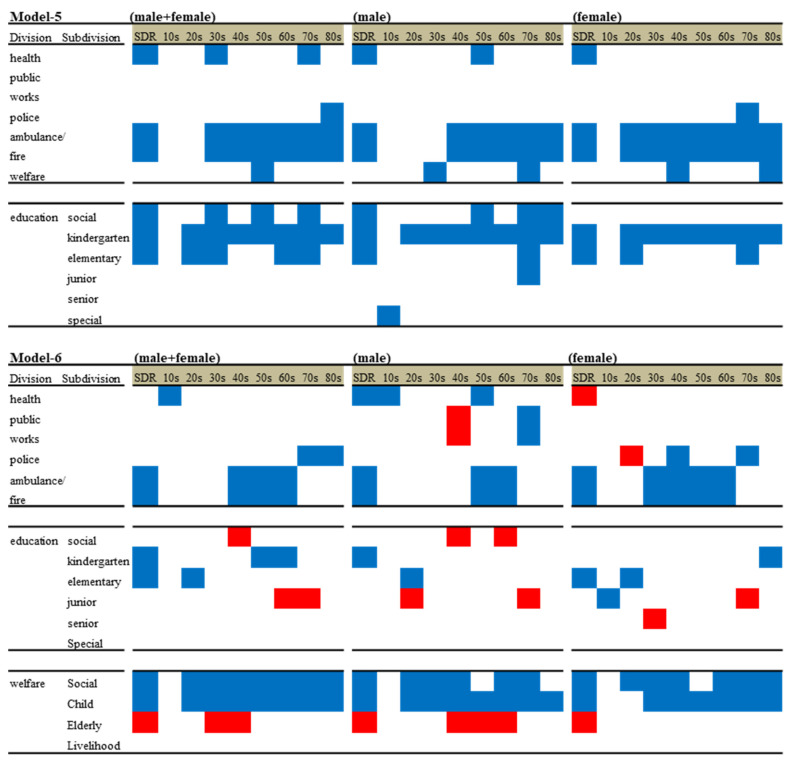
Summary of effects of regional financial expenditure of divisions and subdivisions of education (Model 5) and welfare (Model 6) on suicide mortality of males and females together (M + F), males (M) and females (F) from 2009 to 2018. Blue and red columns, significant factors for decreasing and increasing suicide mortality, respectively.

Hierarchical linear-regression model analysis detected negative relationships between the regional financial expenditure of divisions with education subdivisions and the suicide mortality of males and females together, males, and females disaggregated by age (Figure 6). Public health and ambulance- or fire-service expenditure was negatively related to the SDRs of males and females together, males, and females (Model 3). However, in Model 5, the negative effects of public health expenditure on suicide mortality could not be detected consistently since negative effects were limited against males and females (30s and 70s) and males (50s). The expenditure of ambulance and fire services was negatively related to the suicide mortality of working ages and the elderly, males and females (30s–80s), males alone (40s–80s), and females alone (20s–80s) (Figure 6). 

Regarding the expenditure of education subdivisions, in Model 3, kindergarten and elementary school expenditure was negatively related to the SDRs of males and females together, males, and females. In Model 5, kindergarten school expenditure was negatively related to the suicide mortality of all ages of both males and females except for 10s. Elementary school expenditure was negatively related to the suicide mortality of males and females of parent (20s and 30s) and grandparent (60s and 70s) ages, whereas gender-disaggregated analysis detected limited negative effects on the suicide mortality of males (70s) and females (70s). In Model 3, social-education expenditure was negatively related to the SDRs of males and females together and males alone, but not that of females alone. In Model 5, social education expenditure was negatively related to the suicide mortality of elderly males (50s, 70s, and 80s), but did not affect any female ages. Special school expenditure was related to the suicide mortality of males (10s), but the suicide mortality of females (10s) was not related to any expenditure (Figure 6). 

#### 3.5.2. Effects of Regional Financial Expenditure of Divisions and Subdivisions of Education and Welfare on Suicide Mortality Disaggregated by Ages and Genders (Model 6)

In Models 3 and 4, the negative effect of elementary school expenditure on the SDRs of males and females together, males, and females disappeared due to the additions of the expenditure of welfare subdivisions into the independent valuables (Figure 5). In Model 6, the negative effect of elementary school expenditure on the suicide mortality of males and females together, males, and females was limited in the suicide mortality of males and females in their 20s, males alone, and females alone, but the negative relationships of the suicide mortality of other age-range groups disappeared (Figure 6). The negative effect of kindergarten school expenditure on the SDRs of males also disappeared due to the additions of the expenditure of welfare subdivisions into the independent valuables. In Model 6, the effects of the most dominant factor, kindergarten school expenditure, disappeared in all age ranges of males and females (20s–70s). The negative effects of social-education expenditure disappeared in all ages ranges of males and females together, males, and females, but was positively related to the suicide mortality of males and females (40s) and males alone (40s and 60s). The significant effect of junior high school expenditure on suicide mortality was not detected in any models; in Model 6, junior high school expenditure was negatively related to the suicide mortality of females (10s). 

Regarding welfare subdivision expenditure, in Model 4, social and child welfare expenditure was negatively related to the SDRs of males and females together, males, and females, whereas elderly welfare expenditure was positively related to the SDRs of males and females together, males, and females. In Model 6, a negative effect of child welfare expenditure was detected in the suicide mortality of males and females (20s–80s), males alone (20s–80s), and females alone (30s–80s). A negative effect of social welfare expenditure was also detected in the suicide mortality of males and females (20s–80s), males alone (20s–40s and 60s–70s), and females alone (20s–40s and 60s–80s). Elderly welfare expenditure, on the other hand, was positively related to males and females (30s–40s) and males alone (40s–60s), but was not related to the suicide mortality in the elderly aged population.

## 4. Discussion

From 2009 to 2018, yearly national governmental expenditure was almost equal, whereas yearly regional financial expenditure increased by 18% [30,31,37]. Therefore, total financial expenditure in Japan was shifted to regional governmental expenditure in this decade. A major increase in regional governmental expenditure was composed of the increase in welfare expenditure, but other expenditure divisions remained largely unchanged except for a temporary increase in the expenditure of ambulance and fire services from 2013 to 2015, induced by the Great East Japan earthquake [32].

### 4.1. Effects of Regional Governmental Expenditure on Suicide Mortality Disaggregated by Gender

Considering the expenditure of six divisions of regional governments, the hierarchical linear-regression model detected a characteristic impact between the amounts and ratio of expenditure on suicide mortality [37]. Increasing expenditure ratios of public health and police contribute to decreased suicide mortality, but an increased public-works expenditure ratio elevates suicide mortality. The expenditure ratios of ambulance and fire services, welfare, and education did not affect suicide mortality. Contrary to the expenditure ratios, regarding the expenditure amounts of major divisions, increasing the expenditure of public health, ambulance and fire services, and education reduces suicide mortality, whereas the expenditure amounts of public works, police, and education do not affect suicide mortality. These tendencies were also consistent in analysing the dependent valuables with suicide mortality disaggregated by gender. Both police and ambulance systems play important roles in responding to suicide crises in the community [38]. Education and welfare expenditure also contributes to the prevention of suicide [26,39]. Furthermore, the increasing expenditure of public works on infrastructure is traditionally considered to contribute to the improvement of economic and mental wellbeing via the generation of employment opportunities [37]. These discrepancies between the present results and previous findings suggest that the importance of analysing independent variables by both using the expenditure of the six major divisions and including the more detailed expenditure of subdivisions using hierarchical linear-regression model analysis.

The SLPFS publishes the expenditure of subdivisions of welfare and education [30,31]. Therefore, in order to analyse the effects of regional financial expenditure on suicide mortality in more detail, we added the expenditure of subdivisions of education, which was a significant or negatively effective factor on suicide mortality in Model 3, and the expenditure of subdivisions of education with welfare in Model 4. In Model 3, the negative effects of public health and ambulance- and fire-service expenditure on suicide mortality were not affected by education-subdivision expenditure. Regarding the expenditure of education subdivisions, increasing kindergarten and elementary school expenditure reduces suicide mortality, but other subdivisions of junior and senior high schools and special schools did not affect it. The effects of social education expenditure on suicide mortality were clearly detected in the different responses of males and females since social education was negatively related to suicide mortality of males but did not affect that of females. Social education expenditure is composed of the construction of new and the improvement of existing educational facilities, and the salaries of educational officers for operating these facilities [30,31]. Generally, Japanese females are less resistant to community-based contacts than males are [6,12]. Furthermore, males are more socially affected than females are [40,41], resulting in enriching prefectural social community support programmes, including enlightenment programmes, to prevent male suicide mortality [8,9,12]. Therefore, the expenditure of social education probably reduces male suicide mortality by providing males with contact opportunities with the community to protect from isolation. 

Contrary to Model 3, in Model 4, social and child welfare expenditure was negatively related to the suicide mortality of males and females together, males alone, and females alone. Elderly welfare expenditure, on the other hand, was positively related to suicide mortality, but livelihood welfare expenditure did not affect suicide mortality. The negative effects of elementary school expenditure on suicide mortality of males and females together, males alone, and females alone was removed by the additions of welfare subdivision expenditure. The negative effect of kindergarten school expenditure on the suicide mortality of males was also removed by the addition of welfare subdivision expenditure. In spite of a declining birth rate, the discrepancy of the results of Models 3 and 4 suggests that child care is a more severe burden issue of Japanese people than what our expectations were. Child welfare, kindergarten, and elementary school expenditure probably provides welfare support that is necessary for children and places to accommodate children during working hours, respectively. Japanese women are traditionally forced to play subordinate roles due to limited resources for childcare support and are prone to social isolation due to social childcare demands [6,8,9,12]. Indeed, the enhancement of economic and social participation and the contribution of females reduce the suicide mortality of working-age females, but increase the suicide mortality of working-age males (20–50s), the elderly, and school-age populations [8]. In other words, vulnerable sociopsychological resources for childcare support in Japan negatively affect the work–life–family balance of both females and males [42]. These metamorphoses of the protective effects of education subdivision expenditure on suicide mortality are shown in the fact that the protective effects between social welfare and social education, and between child welfare and kindergarten education probably have a similar impact, but the impact of welfare is stronger than that of education. Therefore, the effects of welfare and education subdivision expenditure are dependent on the life–work–family balance.

It is unexpected that livelihood welfare expenditure did not affect the suicide mortality of any gender groups, since the enhancement and deterioration of social support to individuals with economic strain, and mental and physical disabilities play important roles in reduced and increased suicide mortality in the USA and England, respectively [24,26]. The discrepant effects of livelihood welfare expenditure on suicide mortality between the USA, England, and Japan are possibly dependent upon governmental systems for welfare. In Japan, social welfare is mainly for individuals with several disabilities. When social welfare cannot provide sufficient economic support, livelihood welfare provides additional support for individuals. Furthermore, livelihood welfare is composed a number of subdivisions, including assistances of life, housing, education, medical, pregnancy, and long-term care [43]. Therefore, the expenditure of social welfare is the predominant support system for individuals with several types of disabilities rather than livelihood welfare.

### 4.2. Effects of Regional Governmental Expenditure on Suicide Mortality Disaggregated by Gender and Age

Results using analyses of the effects of regional expenditure of divisions and subdivisions on suicide mortality disaggregated by gender and age were difficult to interpret due to various relations. However, the inhibitory effects of kindergarten and elementary school expenditure on suicide mortality were removed by child welfare expenditure. A survey of local public finance settlement published the expenditure of special, kindergarten, elementary, junior high, and senior high schools, and they were calculated per pupil. The results that education expenditure decreases the suicide mortality of both school-age populations and caregiver populations indicate the importance of social resources and places for children during working hours in the establishment of the life–work–family balance of individuals. Indeed, the suicide-preventive effects of education expenditure were removed by child welfare expenditure. Child welfare expenditure, which was composed of expenditure associated with subsidies for medicine, elimination of waiting lists and allowance, was calculated by the expenditure per individuals younger than 18 years in the survey of local public finance settlements. Against the Japanese social crisis of the declining birth rate and ageing population, according to the Outline of Measures for Society with Decreasing Birth Rate adopted by the Cabinet in 2015, child welfare expenditure has been increasing. Therefore, increasing child welfare expenditure contributes to the improvement of suicide mortality due to a reduction in the burden associated with child care [44]. 

Complicated fluctuations were observed regarding the suicide mortality of school-aged populations. In Model 5, the suicide mortality of males (10s) decreased with increased expenditure of special schools; in Model 6, the suicide mortality of males (10s) decreased by increased public health expenditure but not by that of special schools. The previous literature cannot explain the mechanisms of the complicated suicide mortality of males (10s) influenced by regional expenditure; however, this finding suggests that the countermeasure of physical or mental disabilities in males needs improvement. In Model 5, the suicide mortality of females (10s) was not affected by expenditure; in Model 6, the suicide mortality of females (10s) decreased with increased junior high school expenditure. A previous study reported the suicide mortality of females (10s) is caused by school-related motives, saying that it was negatively related to the minors rate per household, but positively to dual-income household rate [8]. Taken together with previous findings, the present results suggest that the enhancement of a substantial daily communication environment probably plays important roles in the mental health for school-age females. 

Contrary to our expectations, elderly welfare expenditure was positively related to the suicide mortality of working-age populations. The special account of medical care for the elderly and the operating spending of welfare centres for the elderly are more than 80% in elderly welfare expenditure in many prefectures [30,31]. Long-term care assurance reduces the suicide mortality of elderly females (80s), and of both males and females caused by health-related motives, which is most frequent suicide pattern in Japan. In 2017, the means of savings and liability per household were JPY 18 million and JPY 5 million, respectively. The means of savings and liability per household of elderly (older than 70 years old) were JPY 23 million and JPY 1 million, respectively; however, the means of savings and liability per household of populations younger than 40 years olds were JPY 6 million and JPY 11 million, respectively [45]. Therefore, the increasing expenditure of elderly welfare increases the suicide mortality of insolvent generations. In other words, reasonable and evidence-based welfare redistribution is required.

### 4.3. Limitations and Future Developments

Although the present study demonstrated the impact of regional financial expenditure on suicide mortality in Japan, it has several limitations. We analysed the expenditure of the major six divisions according to public finance law on suicide mortality; the demonstrated results indicate that the targeted composition of regional financial expenditure probably defines more the expenditure impact on suicide mortality disaggregated by gender and age. Furthermore, we could not detect the impact of livelihood welfare; however, the most dominant livelihood protection in livelihood welfare includes financial, social, medical, and educational support for economic and socially vulnerable individuals (disabled, elderly, and young individuals). Therefore, to clarify the practical sociopsychological pathomechanisms of suicide, more fragmented regional financial expenditure should be restructured according to targeting, and its impact on suicide mortality disaggregated by gender and ages should be analysed.

## 5. Conclusions

In conclusion, the present study revealed that the importance of the effects of regional financial expenditure on suicide mortality between 2009 and 2018 in Japan, using fixed-effect analysis of hierarchal linear regression with robust standard error. The expenditure of public health, police, ambulance and fire services, welfare, and education contributes to a reduction in suicide mortality, at least in some statistical indicators; however, the expenditure of public works displayed no impact or increased suicide mortality. The most effective expenditure in the major six divisions was that of welfare. In the expenditure of welfare subdivisions, child and social welfare expenditure was effective at achieving a reduction in suicide mortality, but elderly welfare expenditure contributed to increasing suicide mortality. The negative effects of child welfare expenditure reached a wide range of male and female ages, whereas the positive effects of elderly welfare expenditure were limited in the increasing in the working-age population positively, but surprisingly did not affect suicide mortality of elderly populations. Regional financial expenditure is essential for maintaining and operating social welfare systems, and it is difficult to drastically revise expenditure independently constructed via policies by regional governments, in addition to the declining birth rate and ageing population in Japan. Even if the relative increase in elderly welfare expenditure and the relative decrease in child welfare are unavoidable due to the social concerns associated with the declining birth rate and ageing population in Japan, we should explore the rational redistribution of welfare expenditure based on socioeconomic and sociopsychological findings.

## Figures and Tables

**Table 1:** Effects of financial expenditure ratio of six divisions of prefectures and municipalities on suicide mortality.

Divisions	Male + Female				Male				Female			
	β	SE	T Ratio	(*p*)	β	SE	T Ratio	(*p*)	β	SE	T Ratio	(*p*)
Public health	−2.107	0.237	−8.892	**	−2.834	0.398	−7.113	**	−1.447	0.124	−11.627	**
Public works	0.845	0.127	6.626	**	1.263	0.218	5.798	**	0.485	0.073	6.636	**
Police	−0.185	0.028	−6.716	**	−0.304	0.048	−6.260	**	−0.075	0.014	−5.285	**
Ambulance/fire	0.016	0.020	0.820		0.037	0.036	1.036		−0.007	0.012	−0.603	
Welfare	−0.038	0.078	−0.485		−0.090	0.131	−0.691		0.010	0.037	0.254	
Education	0.111	0.129	0.858		−0.084	0.186	−0.452		0.297	0.099	3.018	

β, coefficient; SE, standard error. ** *p* < 0.01 by hierarchal linear-regression model.

**Table 2:** Effects of financial expenditure per capita of six divisions of prefectures and municipalities on suicide mortality.

Divisions	Males and Females				Males				Females			
	β	SE	T Ratio	(*p*)	β	SE	T Ratio	(*p*)	β	SE	T Ratio	(*p*)
Public health	−0.107	0.051	−2.102	*	−0.348	0.079	−4.375	**	0.121	0.040	3.031	**
Public works	0.085	0.237	0.360		0.098	0.358	0.272		0.075	0.129	0.578	
Police	−1.675	2.046	−0.818		−0.401	2.924	−0.137		−2.872	1.443	−1.990	
Ambulance/fire	−5.372	1.027	−5.231	**	−7.604	1.450	−5.246	**	−3.416	0.700	−4.880	**
Welfare	−0.263	0.174	−1.514		−0.421	0.266	−1.581		−0.118	0.091	−1.288	
Education	−2.123	0.309	−6.877	**	−3.279	0.469	−6.985	**	−1.082	0.207	−5.232	**

β, coefficient; SE, standard error. * *p* < 0.05, ** *p* < 0.01 by hierarchal linear-regression model.

## Data Availability

All raw data are available to any persons from Japanese National databases in the Statistics Bureau of the Ministry of Internal Affairs and Communications (SBMIAC), Cabinet Office (CAO) and Ministry of Health, Labour and Welfare (MHLW).
